# A cost-utility analysis of *BRCA1* and *BRCA2* testing in high-risk breast cancer patients and family members in Thailand: a cost-effective policy in resource-limited settings

**DOI:** 10.3389/fpubh.2023.1257668

**Published:** 2023-12-14

**Authors:** Pongtawat Lertwilaiwittaya, Narisa Tantai, Satanun Maneeon, Sophittha Kongbunrak, Nongyao Nonpanya, Anna C. E. Hurst, Varalak Srinonprasert, Manop Pithukpakorn

**Affiliations:** ^1^Department of Medicine, University of Alabama at Birmingham, Birmingham, AL, United States; ^2^Department of Genetics, University of Alabama at Birmingham, Birmingham, AL, United States; ^3^Department of Medicine, Faculty of Medicine Siriraj Hospital, Mahidol University, Bangkok, Thailand; ^4^Siriraj Genomics, Faculty of Medicine Siriraj Hospital, Mahidol University, Bangkok, Thailand; ^5^Department of Pharmacy, Faculty of Medicine Siriraj Hospital, Mahidol University, Bangkok, Thailand; ^6^Siriraj Health Policy Unit, Faculty of Medicine Siriraj Hospital, Mahidol University, Bangkok, Thailand

**Keywords:** genetic testing, BRCA, cost-utility, breast cancer, middle-income, health policy, cost-effectiveness

## Abstract

**Background:**

Screening for germline pathogenic *BRCA1* or *BRCA2* variants (gBRCA) in high-risk breast cancer patients is known to be cost-effective in high-income countries. Nationwide adoption of genetics testing in high-risk breast cancer population remains poor. Our study aimed to assess gBRCA health economics data in the middle-income country setting of Thailand.

**Methods:**

Decision tree and Markov model were utilized to assess cost-utility between the testing vs. no-testing groups from a societal and lifetime perspective and lifetime. We interviewed 264 patients with breast/ovarian cancer and their family members to assess relevant costs and quality of life using EQ-5D-5L. One-way sensitivity, probabilistic sensitivity (Monte Carlo simulation), and budget impact analyses were done to estimate the outcome under Thailand's Universal Health Coverage scheme.

**Results:**

The predicted lifetime cost and Quality-adjusted Life Years (QALY) for those with breast cancer were $13,788 and 10.22 in the testing group and $13,702 and 10.07 in the no-testing group. The incremental cost-effectiveness ratio for gBRCA testing in high-risk breast cancer patients was $573/QALY. The lifetime cost for the family members of those with gBRCA was $14,035 (QALY 9.99), while the no-testing family members group was $14,077 (QALY 9.98). Performing gBRCA testing in family members was cost-saving.

**Conclusion:**

Cost-utility analysis demonstrated a cost-effective result of gBRCA testing in high-risk breast cancer patients and cost-saving in familial cascade testing. The result was endorsed in the national health benefits package in 2022. Other middle-income countries may observe the cost-effective/cost-saving aspects in common genetic diseases under their national health schemes.

## 1 Introduction

Germline genetic testing of *BRCA1* and *BRCA2* (gBRCA) has become an integral part of the current management of breast and ovarian cancer. At the individual level, the genetic results influence patients' eligibility for PARP inhibitors and increased surveillance to reduce contralateral or second primary cancer (1). At the family level, cascade testing expands the opportunities for cancer prevention, both medically and surgically. The importance of a molecular diagnosis led the United States Preventive Services Task Force (USPSTF) to recommend gBRCA in at-risk patients nationally since 2005 (2).

Healthcare systems play a crucial role in the distribution of the resources for genetic risk assessment. Despite an increase in awareness of gBRCA testing in the United States, notably after the “Angelina” effect (3), <5% of USPSTF guideline-eligible women who underwent a mammography 10 years after the recommendation at a university hospital in the US had received gBRCA testing (4). Multiple interventions to reform the healthcare through diversity, equity, and inclusion efforts for at-risk populations have been a decade-long focus. Addressing the financial constraints through the Affordable Care Act (ACA) seemed to increase the uptake of gBRCA in marginalized populations (5). Relaxed testing criteria, from the USPSTF 2005 to the National Comprehensive Cancer Network (NCCN) guideline, is being adopted to expand the high-risk territory (6). To date in the United States, the cost of genetic counseling and testing are being covered under Medicare or Medicaid in most states. Yet, how the federal organization and other major stakeholders enact the inclusion of gBRCA as a national effort remains to be seen.

Globally, the economic evaluation of gBRCA testing had been studied in several settings. The gain in life-years and the cost-effectiveness in gBRCA testing were concurringly productive under each countries' threshold (7). The interventions following detection of pathogenic/likely pathogenic gBRCA variants, including risk-reducing mastectomy (RRM) and risk-reducing salpingo-oophorectomy (RRSO), have been included with difference scenarios in those cost-effectiveness studies (8). Recent work incorporated familial cascade testing to the model, and the cost-effectiveness expanded toward testing in family members (8). However, previous studies were all carried out in high-income countries. With the advancement of techniques for genetic testing that become more widely available with affordable cost, it appears to be possible for middle- and low-income countries to adopt the practice, more or less similarly to high-income countries. This study aims to report data from an upper-middle income country and highlight the health-economic analysis that rationalized the enrollment of gBRCA testing in high-risk women with breast cancer and their families in Thailand.

## 2 Materials and methods

### 2.1 Model development

**Figure 1:**
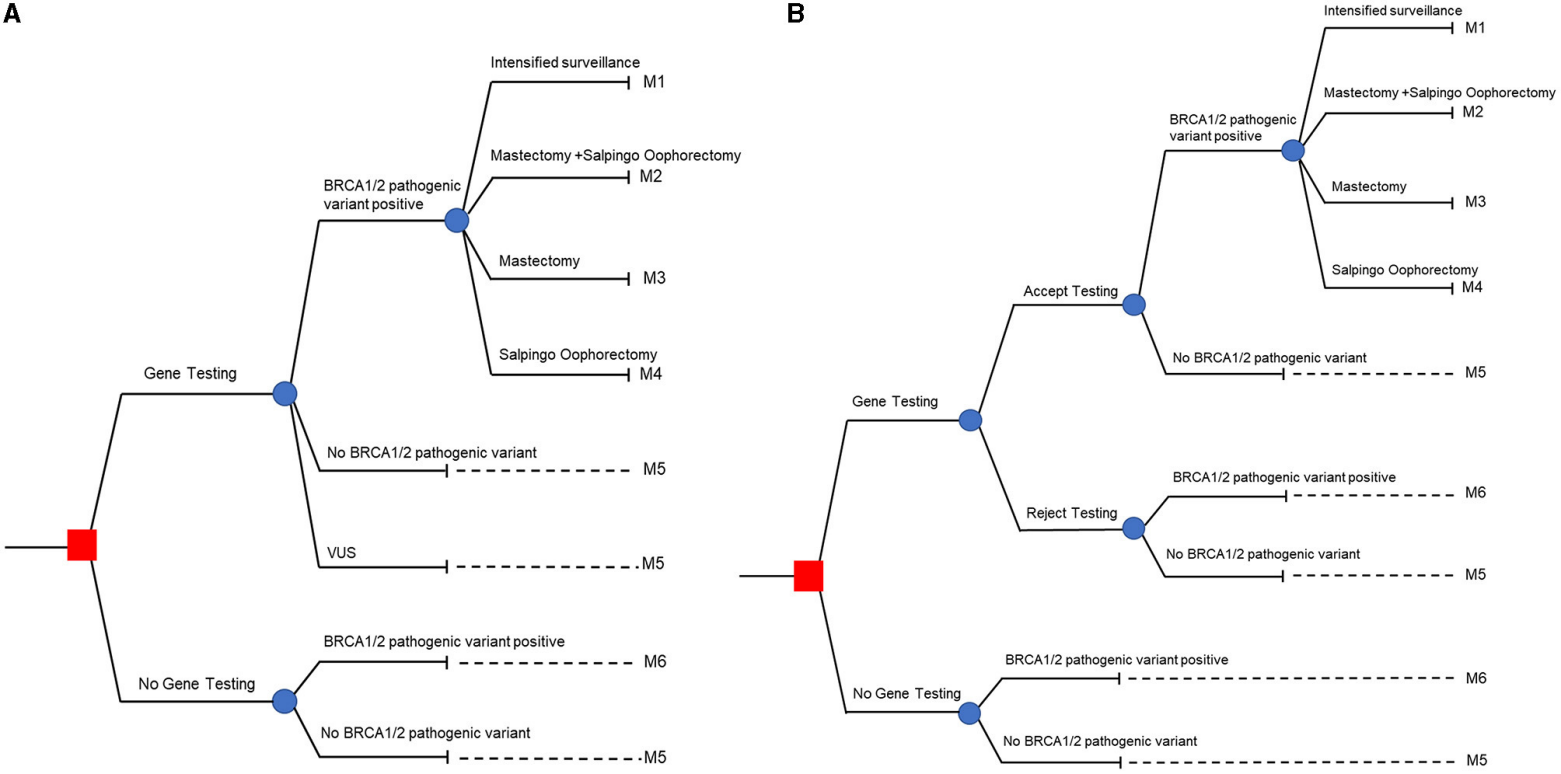
Hybrid decision tree and Markov models. **(A)** Represents patient's perspective, and **(B)** represents family member's perspective.

A model to assess the efficacy and cost of applying genetic testing followed by subsequent management strategies based on gBRCA status was jointly designed, in a stakeholder meeting, by the authors and seven national experts from multiple specialties related to care for the condition (Figure 1A). It was decided to study the population with high-risk breast cancer defined by the 2019 NCCN guidelines for genetic/familial high-risk assessment of breast and ovarian cancers. The model was a hybrid decision tree and Markov model, beginning with the decision of uptaking vs. not uptaking gBRCA testing (labeled with a red square in Figure 1). Each decision was followed by possible outcomes from the test including gBRCA pathogenic/likely pathogenic variant (P/LP), absence of P/LP variant, and Variant of Uncertain Significance (VUS), each of which harbored different carcinogenesis risk and transitional probability in Markov model fashion. In the probands with P/LP variants, choices of intensified surveillance per breast cancer treatment protocol, risk-reducing mastectomy (RRM), and/or risk-reducing salpingo-oophorectomy (RRSO) were counseled and carried through. Different uptakes following the post-test genetics counseling session in the probands led to the probability of entering management stratification in the decision tree and subsequent Markov models designated as M1, M2, M3, and M4 illustrated in Figure 1A. Probands who tested negative for P/LP gBRCA variants were advised to resume routine cancer surveillance and enter the Markov model as the M5 category. Patients with VUS in gBRCA were counseled regarding reclassification of potential future variants and managed individually. The VUS group entered the Markov model as the M5 category as risk-reducing surgery was not recommended and the risk of carcinogenesis was converged toward the negative P/LP group. In the no gBRCA testing decision group, patients who did not harbor P/LP gBRCA variants entered the Markov model via M5 while the probands who tested positive for P/LP gBRCA variants entered the Markov model via M6.

For family members, we used the decision model shown in Figure 1B. The decision tree started with the uptaking vs. not uptaking familial cascade when targeted testing after the P/LP gBRCA proband is established (labeled as red square in Figure 1B). Family members were counseled and provided the option of accepting or declining cascade testing. Targeted sequencing performed in this circumstance, for those accepting the test, led to potential outcomes only with positive and negative specific P/LP gBRCA variants. As in the patient's perspective model, family members with P/LP gBRCA had the choice to opt in for intensified surveillance, RRM, and/or RRSO and proceeded to Markov model via the M1-M4 categories. Family members with negative targeted testing advanced to the Markov model via the M5 category. Family members who declined testing and family members in the not uptaking decision scenario who would have tested positive for P/LP gBRCA progressed toward the Markov model via M6. Lastly, family members who declined testing and family members in the not uptaking decision scenario who would have tested negative for P/LP gBRCA moved along the Markov model via the M5 subgroup.

Overall, the decision tree model stratified the risk of breast and ovarian cancer development based on the natural history and preventative measures taken in patients and their family members in to six groups: M1-M6. M1, M2, M3, and M4 are those who harbor P/LP gBRCA and opted in for a intensified surveillance program, both RRM and RRSO, RRM only, and RRSO only. M5 refers to the group of patients or family members of P/LP gBRCA probands whose genetic test yielded negative or VUS. Note that we do not offer risk-reducing surgery to patients with VUS results, and that the risk of breast/ovarian tumorigenesis in the VUS group was presumed to be on par with the negative result. M6 represented patients or family members with presumably undiagnosed P/LP gBRCA and would follow the natural history of gBRCA patients without risk-reducing intervention other than the standard breast cancer treatment/surveillance in the proband.

#### 2.1.1 Markov model

**Figure 2:**
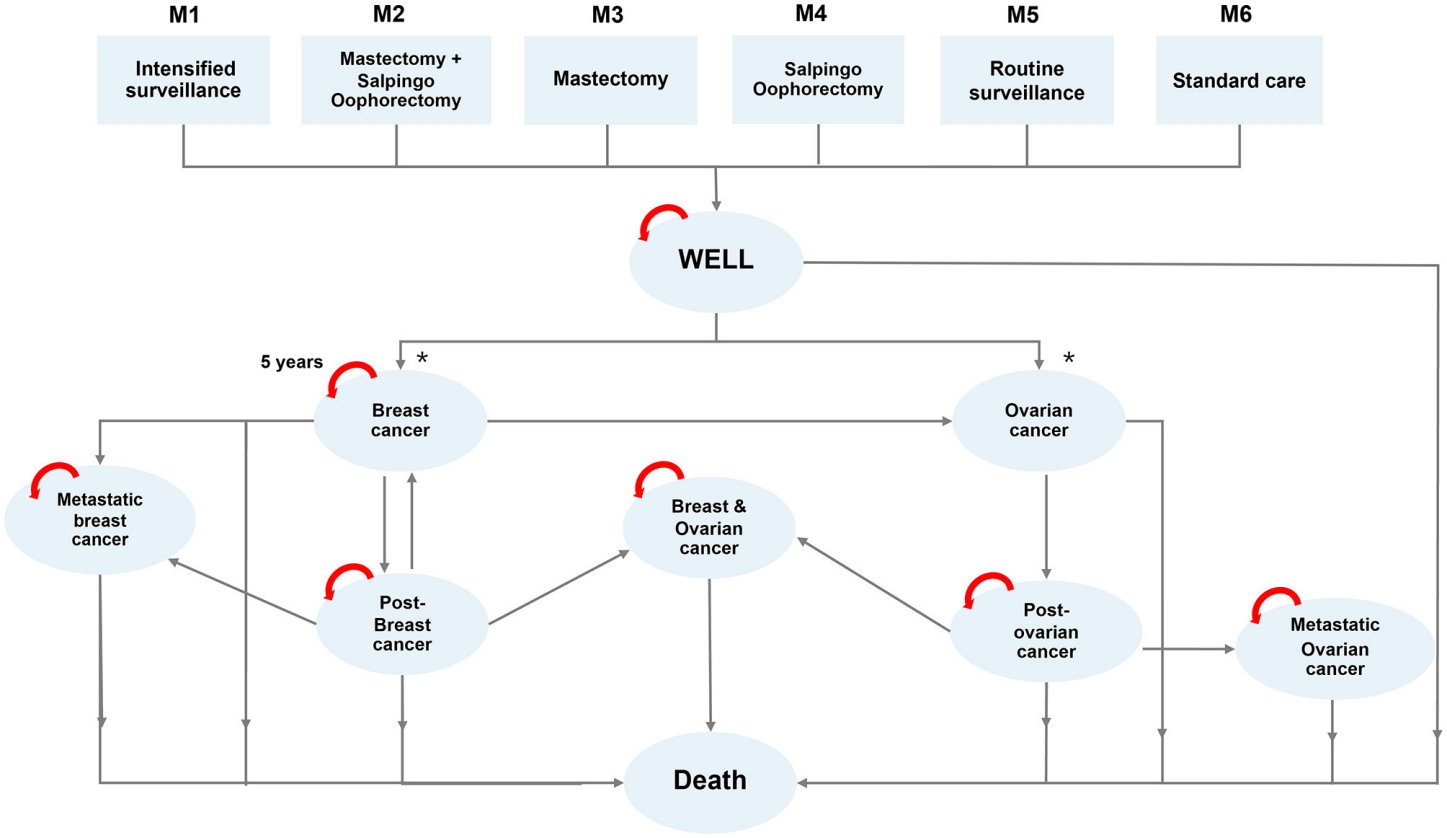
Markov model demonstrating transition of 6 different risk categories (M1-M6) to different states of health.

A Markov model was designed to estimate relevant health outcomes and costs using a lifetime horizon from the societal perspective. Our model accounted for the possible consequences with both breast cancer and ovarian cancer development, with post-treatment and progression (metastasis) as different health states (Figure 2). We set the entry age to be 45 years old for both patient and family members, established a cycle length of 1 year, and followed the patient throughout their lifetime (until death). The entry age in this study was derived from NCCN guideline 2019 v.2 and the observed age of population in our previous study. The discount rate in this study was set at 3%.

### 2.2 Input data

#### 2.2.1 Internal data

The study protocols were approved by the Siriraj Hospital IRB protocol COA approval numbers 656/2019, 631/2019, and 508/2020. The study population included all breast cancer patients who received gBRCA testing and breast cancer treatment at Siriraj Hospital, a large medical school accepting referrals from all provinces in the country (*n* = 257). Family members (*n* = 7) were recruited from those who underwent targeted-variants familial cascade testing at the medical genetics clinic at Siriraj Hospital. Direct medical and non-medical costs were obtained by interviewing the study population and from the hospital database. Utilities were retrieved by interviewing using the ED-5Q-5L questionnaire. A total of 264 patients and family members in different health states were recruited for interviewing (Table 1).

**Table 1:** Summary of input parameters (transitional probability, cost, and utility) for base-case analysis.

**Variable(s)**	**Distribution**	**Mean**	**SE**	**Reference**
**Transitional probability**				
Rate of pathogenic/likely pathogenic gBRCA detection in high-risk breast cancer women	Beta	0.1860	0.0186	Internal data from Siriraj Genomics (9)
Rate of Variant of Unknown Significance (VUS) detection in high-risk breast cancer women	Beta	0.0631	0.0063	
Rate of targeted variants testing uptake in family members within 1 year	Beta	0.1643	0.0164	
Rate of positive familial cascade testing within 1 year	Beta	0.5	N/A	
**Rate of risk-reducing surgery uptake at 1-year follow up in proband with P/LP gBRCA**				
RRM and RRSO	Beta	0.1897	0.0190	Internal data from Siriraj database
RRM	Beta	0.1034	0.0103	
RRSO	Beta	0.1897	0.0190	
**Rate of risk-reducing surgery uptake at 1-year follow up in family member with P/LP gBRCA**				
RRM and RRSO	Beta	0	0	Internal data from Siriraj database
RRM	Beta	0	0	
RRSO	Beta	0.2727	0.0273	
**Risk of breast and/or ovarian cancer development transitional probability in different prevention strategies**				
Rate of breast cancer development in P/LP gBRCA with intensified surveillance	Beta	0.0364	0.00364	(10)
Rate of breast cancer development in RRM + RRSO	Beta	0.0037	0.00037	(11)
Rate of breast cancer development in RRM	Beta	0.0072	0.00072	(11)
Rate of breast cancer development in RRSO	Beta	0.0178	0.00026	(12)
Rate of breast cancer development in negative gBRCA with intensified surveillance	Beta	0.0072	0.00072	(10)
Rate of ovarian cancer development in P/LP gBRCA with intensified surveillance	Beta	0.0128	0.01282	(13)
Rate of breast cancer development in RRM + RRSO	Beta	0.0018	0.00065	(14)
Rate of breast cancer development in RRM	Beta	0.0036	0.00005	(14)
Rate of breast cancer development in RRSO	Beta	0.0027	0.00059	(12)
Rate of ovarian cancer development in negative gBRCA with intensified surveillance	Beta	0.0006	0.00062	(15)
**Variables used in state of health change in Markov model**				
Rate of breast cancer metastasis	Beta	0.0211	0.0021	(16)
Rate of breast cancer recurrence in 1-5 yr	Beta	0.2432	0.0243	(17)
Rate of breast cancer recurrence in 6-10 yr	Beta	0.0749	0.0075	(17)
Rate of breast cancer recurrence in 11-15 yr	Beta	0.0383	0.0038	(17)
Rate of breast cancer recurrence after 16 yr	Beta	0.0445	0.0045	(17)
Rate of ovarian cancer development in breast cancer patient	Beta	0.0007	0.0001	(18)
Rate of breast cancer development in ovarian cancer patient	Beta	0.0040	0.0004	(19)
Mortality rate of breast cancer patient in the first year	Beta	0.1500	0.0230	(20)
Mortality rate of breast cancer patient in the 2-3 year	Beta	0.1391	0.0383	(20)
Mortality rate of breast cancer patient in the 4-5 year	Beta	0.1091	0.0383	(20)
Mortality rate of breast cancer patient in the 6-10 year	Beta	0.0758	0.0076	(17)
Mortality rate of breast cancer patient in the 11-15 year	Beta	0.0390	0.0039	(17)
Mortality rate of breast cancer patient after 16 year	Beta	0.0465	0.0047	(17)
Mortality rate of breast and ovarian cancer patient in the 1-5 year	Beta	0.0195	0.0510	(21)
Mortality rate of breast and ovarian cancer patient after 6 year	Beta	0.0069	0.0612	(21)
**Medical cost**				
**Direct medical costs**				
Cost of gBRCA test with next generation sequencing (USD)	Gamma	666.88	133.375	Internal data from Siriraj Genomics
Cost of targeted sequencing in family member (USD)	Gamma	78.13	**78.125**	
**Outpatient medical cost (USD/year)**				
Breast cancer	Gamma	902.31	2,310.5	Internal data from Siriraj database
Post-breast cancer	Gamma	350.13	1,258.5	
Metastatic breast cancer	Gamma	1,824.50	4,539.344	
Breast cancer + ovarian cancer	Gamma	1,331.81	3,106.219	
Ovarian cancer	Gamma	448.63	1,153.69	
Post-ovarian cancer	Gamma	199.78	954.56	
Metastatic ovarian cancer	Gamma	1,193.22	2,688.53	
**Inpatient medical cost (USD/year)**				
Breast cancer	Gamma	2,354.44	2,527.09	Internal data from Siriraj database
Post-breast cancer	Gamma	2,289.41	3,615.91	
Metastatic breast cancer	Gamma	3,469.19	5,828.94	
Breast cancer + ovarian cancer	Gamma	3,533.81	4,247.16	
Ovarian cancer	Gamma	2,543.50	1,648.91	
Post-ovarian cancer	Gamma	2,515.59	2,139.38	
Metastatic ovarian cancer	Gamma	4,087.28	5,087	
**Direct non-medical costs (USD/visit)**				
Transportation cost	Gamma	17	27.97	Interview
Meal cost for patient/family	Gamma	3.97	8.94	
Accomodation cost	Gamma	3.22	21.97	
Opportunity cost for family member	Gamma	19.53	43.38	
**Only for first year: supportive equipment and house renovation (USD/year)**				
Well	Gamma	126.19	321.28	Interview
Breast cancer	Gamma	282.72	1,201.28	
Post-breast cancer	Gamma	3.13	10.16	
Metastatic breast cancer	Gamma	209.59	631.13	
Breast cancer + ovarian cancer	Gamma	96.88	247.56	
Ovarian cancer	Gamma	97.09	130.09	
Post-ovarian cancer	Gamma	5.22	15.63	
Metastatic ovarian cancer	Gamma	260.41	247.56	
**From first and following years: supplements, caregiver, private clinic (USD)**				
Breast cancer	Gamma	1,289.56	2,359.03	Interview
Post-breast cancer	Gamma	466.47	1609	
Metastatic breast cancer	Gamma	1,980.34	3,954.06	
Breast cancer + ovarian cancer	Gamma	992.50	2,025.72	
Ovarian cancer	Gamma	756.78	1,469.97	
Post-ovarian cancer	Gamma	223.78	373.06	
Metastatic ovarian cancer	Gamma	3,250.00	5,629.16	
**Utility values**				
Well	Beta	0.89	0.13	Interview
Breast cancer	Beta	0.84	0.18	
Post-breast cancer	Beta	0.90	0.09	
Metastatic breast cancer	Beta	0.80	0.16	
Breast cancer + ovarian cancer	Beta	0.86	0.11	
Ovarian cancer	Beta	0.76	0.18	
Post-ovarian cancer	Beta	0.88	0.13	
Metastatic ovarian cancer	Beta	0.71	0.39	

RRM, Risk-reducing mastectomy; RRSO, Risk-reducing salpingo-oophorectomy; USD, US Dollar.

#### 2.2.2 Cost

##### 2.2.2.1 Direct medical cost

Sequencing cost: All genetic testing was performed at the Siriraj Genomics Laboratory with the next-generation sequencing technology. The cost of genetic testing for probands through the panel was 21,340 THB ($666.88) and the cost of familial cascade testing (targeted variant testing by Sanger sequencing) was 2,500 THB ($78.13) in 2021. Note that, in our study, the genetic testing utilized was next-generation sequencing (NGS) based on a recent study that suggested an absence of significant founder variants in a Thai population (9). Deletion/duplication analysis was integrated as part of the bioinformatics pipeline. A multiple Ligation-Dependent Probe Amplification technique was used to screen and confirm the presence of large copy number variation.

Cancer care cost: We reviewed in-hospital care costs for included patients who received care at Siriraj Hospital from the hospital database between 2017-2021 based on ICD-10 that matched eight states of health. Out-of-hospital costs for each state of health were obtained through interview and listed in Table 1.

##### 2.2.2.2 Indirect medical cost

The indirect costs were obtained by interviewing patients in different health states who received care at Siriraj Hospital during October 2020—March 2021. The interview was carried out by trained research assistants using a standardized questionnaire collecting data according to guidelines recommended for health technology assessment in Thailand. Inquired data included transportation costs, meals during hospital visits for patient/family member, and accommodation costs for patients and family members incurred while receiving cancer treatments at the hospital for all health states of interest. The number of visits to the hospital for cancer treatments was also inquired. The cost of supportive equipment in the first year of diagnosis together with cost of caregivers, supplements, and private clinics in the long term was also obtained through interviewing the patients and families. Moreover, costs of opportunity lost for family members and informal care givers were also included. The average indirect cost is shown in Table 1.

### 2.3 Transitional probability

The rate of P/LP and VUS gBRCA in high-risk breast cancer patients per NCCN 2019.2 recommendation among a Thai population were 18.6% and 6.31% respectively (*N* = 301) (9).

Decisions on Preventive measures were reviewed from a total of 58 P/LP gBRCA patients from the medical genetics clinic at Siriraj hospital between February 2017 and March 2021 (allowing at least 1-year follow up after the date of genetic testing). 11 patients (18.97%) received both RRM and RRSO, 16 patients (27.59%) received RRSO, and six patients (10.34%) received RRM. The rest opted in for intensified surveillance.

There were a total of 280 first-degree relatives for the included P/LP gBRCA proband (average of five first-degree relatives per proband). Only 46 family members (16.43%) had their cascade testing done at the 1-year mark after the date of the proband's genetic test. There were 11 family members (23.91%) who tested positive for targeted sequencing. Amongst the 11 family members with positive familial cascade testing, three (27.27%) underwent RRSO. The rest opted in only for intensified surveillance. All the values are demonstrated in Table 1.

### 2.4 Data for budget impact analysis

The Incidence and prevalence of breast cancer in Thailand were 22,158 cases/year and 76,440 cases in 2020–2021 respectively (22). The rate of high-risk breast cancer based on NCCN guideline 2019 v.2 amongst all breast cancer patients was estimated to be 30% per national experts' consensus. The number of new high-risk breast cancer patients that would qualify for gBRCA testing was 6,648 patients annually (22,158 x 30%), while the number of existing high-risk breast cancer patients was calculated to be 22,932 (76,440 x 30%). The rate of p/lp gBRCA in high-risk breast cancer women in Thailand was 18.6% (9) and the acceptance rate of familial cascade testing from our internal data was 16.43%. It was estimated that one patient would have four first-degree relatives in the age-group that would be eligible for familial cascade testing. Overall, the number of newly diagnosed p/lp gBRCA high-risk breast cancer patients was estimated to be 1,237 (6,648 x 18.6%) annually, while the number of their positive p/lp gBRCA family members accepting testing would be 406 (1,237 × 16.43% × 4 × 50%). The national's sequencing throughput were estimated to be 3,500 samples per year and was projected to increase by 55% annually.

## 3 Results

Cost-utility analysis results are summarized in Table 2. The incremental cost-effectiveness ratio (ICER) calculated for the life-time cost included genetics testing minus the cost for no genetics testing divided by the quality-adjusted life years (QALY) difference between genetics testing and no genetics testing. Offering gBRCA testing in high-risk breast cancer patients resulted in an ICER of $573 per one QALY gained. Offering familial cascade testing in the family members was cost saving at an ICER of $4,200/QALY.

**Table 2:** Outcome estimation from a model-based cost utility analysis comparing genetic testing vs. no genetic testing in the high-risk patient and the family member with P/LP gBRCA.

	**Genetic testing**	**No genetic testing**	**Increment**
**Proband**			
Cost (USD)	$13,788 (12,838–14,737)	$13,702 (12,775–14,630)	$86 (44–126)
QALY	10.22 (10.14–10.30)	10.07 (9.99–10.16)	0.15 (0.14–0.15)
ICER			$573/QALY (435–723)
**Family**			
Cost (USD)	$14,035 (13,120–14,950)	$14,077 (13,163–14,990)	–$42 –(36–47)
QALY	9.99 (9.91–10.07)	9.98 (9.90–10.06)	0.01 (0.012–0.014)
ICER			–$4,200/QALY –(3,560–9,112)

**Figure 3:**
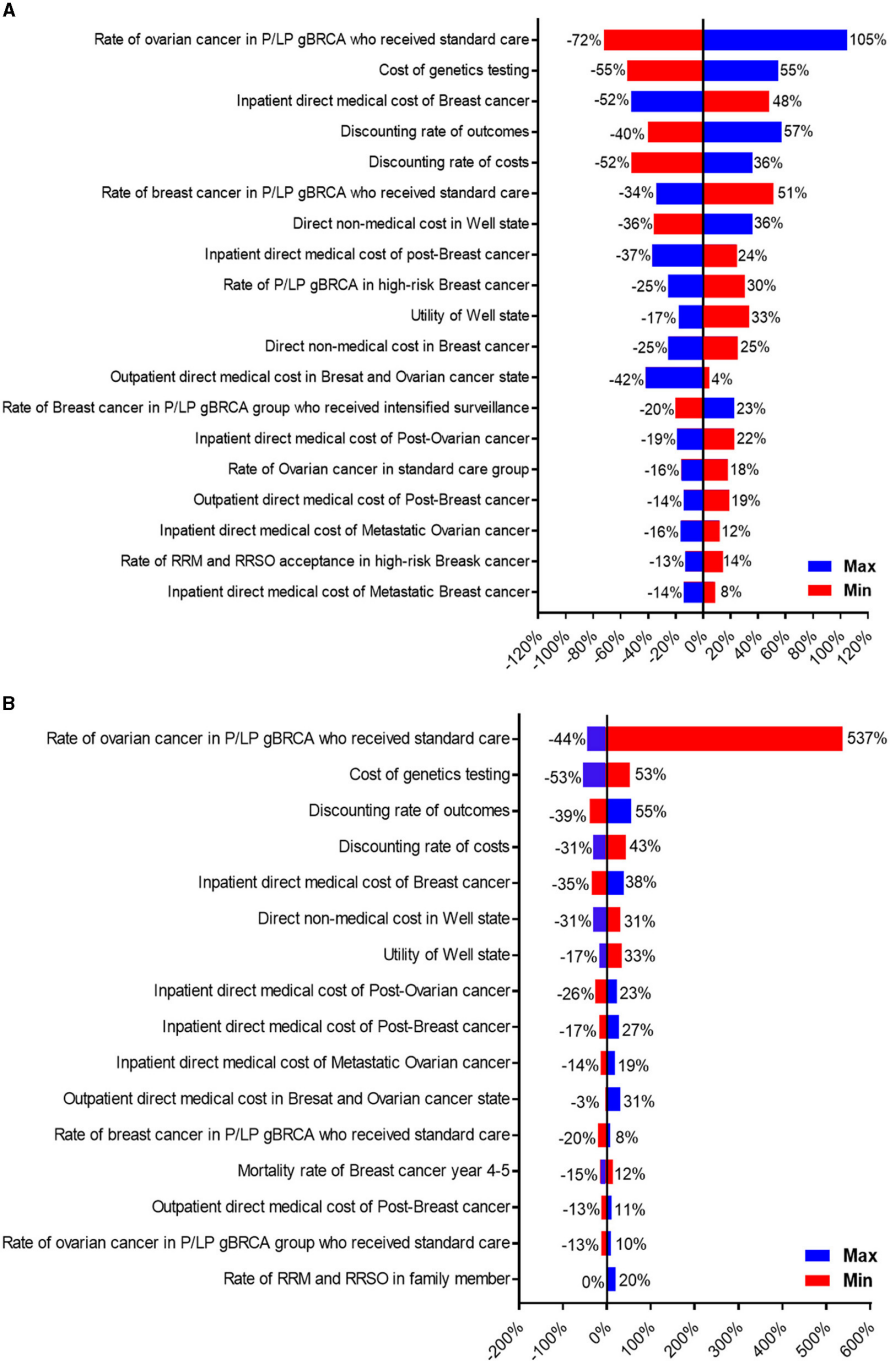
Tornado diagram illustrating one-way sensitivity analysis of variables toward ICER. **(A)** Represents patient's perspective, and **(B)** represents family member's perspective.

One-way sensitivity and probabilistic sensitivity analyses were also performed to determine the potential effects of each factor. Tornado diagrams from one-way sensitivity analyses for patients and family members were shown in Figures 3A, B. From the patient's perspective (Figure 3A), the three most impactful variables toward ICER were the percentage of P/LP gBRCA patients who opted in for intensified surveillance for ovarian cancer instead of RRSO, the sequencing cost, and the inpatient treatment cost of breast cancer. From the family member's perspective (Figure 3B), the most impactful variable remained to be the probability of intensified surveillance (positive) for ovarian cancer, followed by the cost of genetics test and the discounting rate for outcome.

The probability that implementing genetics testing in high-risk breast cancer women at the willingness-to-pay threshold (WTP) at $5,000/QALY (Thailand's threshold) will be cost-effective was 0.84. The probability of performing family cascade testing at the willingness-to-pay threshold at $5000/QALY will be cost-effective was 0.83.

The budget impact analysis is shown in Table 3. The estimated impact on the national budget in endorsement of gBRCA testing for high-risk breast cancer patients under the Universal Health Care coverage scheme in Thailand on the first year was US$ 2.34 million and the estimated 5-year budget impact assuming that genetics testing capacity will increase by 55% annually was US$ 34.69 million.

**Table 3:** 5-year Budget impact analysis between no genetics testing vs. BRCA testing followed by preventive strategies.

**Strategy**	**Annual budget (million USD)**					
	**1st year**	**2nd year**	**3rd year**	**4th year**	**5th year**	**Total**
**Strategy 1: no genetic testing**						
Patient's cancer care cost	0.66	1.94	3.78	6.69	11.25	24.31
Family's cancer care cost	0.5	1.38	2.81	5.09	8.66	18.41
Total	1.16	3.31	6.59	11.78	19.91	42.75
**Strategy 2: genetics testing and subsequent prevention measures**						
Testing cost—patient	2.34	3.63	5.59	8.69	13.47	33.72
Testing cost—family	0.03	0.06	0.09	0.13	0.19	0.47
Total testing cost	2.38	3.66	5.69	8.81	13.66	34.22
Patient's cancer prevention and treatment cost	0.63	1.66	3.34	6.03	10.19	24.84
Family's cancer prevention and treatment cost	0.5	1.38	2.78	5.06	8.59	18.28
Total cancer prevention and treatment cost	1.13	3.03	6.13	11.06	18.78	40.13
Total	3.5	6.69	11.81	19.88	32.44	74.34
Budget impact	2.34	3.38	5.25	8.09	12.56	34.59

## 4 Discussions

This study pioneered health economics data regarding the implementation of gBRCA genetic testing in a high-risk population in Thailand, a middle-income country. An addition of gBRCA testing in high-risk breast cancer patients defined by NCCN guideline 2019 v.2 was cost-effective (ICER 573 USD/QALY) which is below the national WTP of 5,000 USD/QALY. Expanding the intervention to familial cascade testing of P/LP gBRCA was proven to be cost-saving. The result of this study was presented to the National Health Security Office committee and received strong support. An addition of gBRCA testing in high-risk breast cancer patients and their families was therefore endorsed to be reimbursable under Universal Healthcare Coverage and all other health schemes in Thailand in 2022.

Data regarding cost for health care utilization from middle- and low-income countries have been underrepresented in the medical literature. It is intriguing to postulate whether the health economics of genetic services could be more or less cost-effective in resource-limited settings. The cost of clinical genetic services, cancer treatment (both direct and indirect), and preventative strategies in P/LP gBRCA were the main driving factors in this study. When compared to the clinical encounter cost in high-income countries, represented by Australia (8), it appeared that conducting a clinical genetic service in Thailand would be less costly. The sequencing cost in Thailand was lower both in next-generation sequencing ($666 vs. $876) and targeted sequencing ($78 vs. $168). The cost of cancer prevention via surgery once diagnosed with P/LP gBRCA in Thailand was also substantially less costly: $1,239 vs. $11,378 for RRM (8) and $631 vs. $6,293 for RRSO (8) (the cost of interventions in Thailand discussed were from retrieved from a medical school). When looking at the treatment cost, the direct medical cost of breast cancer care for the first year in Thailand was also cheaper ($3,256.75 vs. $17,892). Overall, the cost of implementing gBRCA testing in Thailand appeared to be significantly lower when compared to high-income countries, which may be driven to a certain extent by the lower overall healthcare costs. Provided with the innovative technique with a lower price for gBRCA testing, the lower healthcare cost in other low- to middle-income countries may very well support the cost-effectiveness of genetic services in each country and should be further explored.

The rationale behind a highly cost-effective result in this study might have been more than just differences in healthcare costs. How this study distinguishes itself from previous models was through an addition of transition to ovarian cancer state and prioritization of transitional probability data from an Asian population. The addition of ovarian cancer in the Markov model involved the RRSO into the equation, which could have enhanced the cost-utility when compared to previous models where only breast cancer was formulated as RRSO and proven to be cost-effective (7). Moreover, the higher rate of gBRCA pathogenicity (18.6% vs. 15%) (8) vs presumptive 10% (23, 24) was exploited in our decision tree in accordance with a recent publication which suggested a higher prevalence in Asian patients who fulfilled NCCN testing criteria (9). The higher rate of gBRCA may be partly explained by fewer environmental factors, including obesity, in Asian populations. The adoption of this model in countries with high prevalence of gBRCA pathogenicity may reproduce the cost-utility we reached and is encouraged.

Looking at the sensitivity analysis, it appears that several interventions could strengthen the cost-effectiveness in implementing gBRCA testing in Thailand. The most important factor toward ICER from the patient's perspective was RRSO (Figure 3A). Our RRSO adoption rates (both patients and family members) were significantly lower when compared to previous studies (8). Our lower local surgical adoption rate might be influenced by the cultural differences and the study design, as the patients might not have decided to pursue surgical strategy at 1-year follow up after molecular diagnosis. The promotion of risk-reducing surgery, especially RRSO, may result in a higher risk-reducing surgery rate and would ultimately escalate the ICER toward being more cost-effective. This may contradict a recent long-term study (25) that suggested a lower RRSO adherence led to an improvement in ICER, as the cost of cancer prevention in our country is remarkably cheaper which encourages cancer prevention.

Additionally, achieving a higher test volume once publicly utilized would drop the cost of gBRCA sequencing in half. The effect of economy of scale was discussed with the national experts and under certain scenarios (e.g., 40% reduction in sequencing cost and the national throughput reached a cap), the annual budget impact would gradually decrease and we might observe a cost-saving aspect of genetics testing endorsement. The in-patient treatment cost of breast cancer variability, though, was a dynamic topic of discussion. New treatment modalities in the gBRCA group, such as PARP inhibitors, would tremendously affect the total cost of care. If targeted therapy could be reimbursed under the universal healthcare coverage in Thailand, the cost of in-patient treatment would have changed. However, it is difficult to predict the changes in cost-utility outcomes until a proper model is conducted as better clinical outcomes and quality of life might accompany the higher cost of treatment. Nevertheless, our team anticipated a trend toward the addition of gBRCA testing being more cost-effective in the future, with a higher uptake rate of genetic testing in high-risk breast cancer patients. Meanwhile, the cost effectiveness of including the novel promising treatment might be possible with the implementation of other policies.

## 5 Model limitation

Decision tree and Markov model utilization in health economics evaluation has to evolve overtime to reflect recent advancements in the management of gBRCA patients. In this study, our model incorporated the potential result of “Variant of Uncertain Significance”, which has been recommended by the ACMG since 2015. We also took other malignancy/metastasis into account as previously discussed. This model does not incorporate the impact of medical interventions in the prevention of breast/ovarian cancer, including selective estrogen modulators and aromatase inhibitors, which were not approved for breast cancer prevention in Thailand at the time we conducted the study. Future models that integrate the use of pills as a choice of cancer prevention and the chance of other cancer development including prostate cancer and pancreatic cancer is warranted. Our model also did not address familial cascade testing in the younger generation (children) whose risk-reducing interventions' adoption rate might have differed and changed the results given the metabolic risk from post-surgical ovarian failure. A recent population-based screening model suggested that the incremental cost to adopt genetics testing might be less when done in younger populations (26). Future model expanding into this younger population may observe a lower ICER and is encouraged with caution as stated above.

## 6 Mass implementation in a resource-limited country

A steep global increase in sequencing throughput has played an important role in the assimilation of genetic testing in the prevention of many diseases at the population level. These include cystic fibrosis and spinal muscular atrophy detection via newborn screening, prenatal thalassemia screening in high-prevalent countries, and certain pharmacogenomic testing (e.g., HLA-B^*^5801 before allopurinol prescription). In recent years, genetic testing in germline *BRCA1* and *BRCA2* has become a center of discussion. The benefit in recommending earlier breast cancer screening (starting at 25 years of age per NCCN guidelines compared to 50 years in average-risk women per USPTF recommendation) and preventive measures (estrogen receptor modulator and risk-reducing surgery) influenced the decision to make *BRCA1* and *BRCA2* two of the first 56 genes to be reported as important secondary findings in clinical exome/genome sequencing per ACMG (27). To our knowledge, amidst the consensus of population-wide testing vs. high-risk group testing, gBRCA testing remains far from being widely adopted at the public health level, with a few countries nearly reaching a nationwide policy. As Thailand launched the adoption of gBRCA for high-risk breast cancer patients as part of their government-sponsored universal healthcare coverage, we believe that other countries with higher willingness-to-pay thresholds e.g., Japan (USD$20,000-50,000/QALY), UK (USD$25,000-$37,500/QALY), Australia (USD$35,000/QALY), and the US (USD$50,000-100,000/QALY), or other countries within the same economic tier and with similar healthcare policies to Thailand (e.g., Argentina, Brazil, Malaysia, Mexico, Peru, South Africa, and Turkey) could consider their capability to launch a nationwide policy in the near future.

## 7 Conclusions

Cost-utility analysis demonstrated a highly cost-effective outcome of gBRCA testing in high-risk breast cancer patients in Thailand. Expanding the test to the proband's family members was cost-saving from a societal perspective. Other resource-limited countries may benefit from the endorsement of genetics testing for high-risk breast cancer patients under their national health care plan.

## Data availability statement

The original contributions presented in the study are included in the article/supplementary material, further inquiries can be directed to the corresponding authors.

## Ethics statement

The studies involving humans were approved by Siriraj Institutional Review Board, Faculty of Medicine Siriraj Hospital, Mahidol University, Thailand. The studies were conducted in accordance with the local legislation and institutional requirements. The participants provided their written informed consent to participate in this study.

## Author contributions

PL: Conceptualization, Data curation, Formal analysis, Investigation, Methodology, Validation, Visualization, Writing—original draft. NT: Data curation, Formal analysis, Investigation, Methodology, Writing—review & editing. SM: Data curation, Formal analysis, Investigation, Methodology, Writing—review & editing. SK: Data curation, Formal analysis, Investigation, Methodology, Writing—review & editing. NN: Validation, Visualization, Writing—review & editing. AH: Validation, Writing—review & editing. VS: Conceptualization, Data curation, Formal analysis, Funding acquisition, Investigation, Methodology, Project administration, Resources, Supervision, Validation, Writing—review & editing. MP: Conceptualization, Funding acquisition, Investigation, Methodology, Project administration, Resources, Supervision, Validation, Writing—review & editing.

## References

[1] Yoshida R. Hereditary breast and ovarian cancer (HBOC): review of its molecular characteristics, screening, treatment, and prognosis. Breast Cancer. (2021) 28:1167–80. 10.1007/s12282-020-01148-2 32862296 PMC8514387

[2] U.S. Preventive Services Task Force. Genetic risk assessment and BRCA mutation testing for breast and ovarian cancer susceptibility: recommendation statement. Ann Intern Med. (2005) 143:355–61. 10.7326/0003-4819-143-5-200509060-00011 16144894

[3] Liede A Cai M Crouter TF Niepel D Callaghan F Evans DG. Risk-reducing mastectomy rates in the US: a closer examination of the Angelina Jolie effect. Breast Cancer Res Treat. (2018) 171:435–42. 10.1007/s10549-018-4824-9 29808287 PMC6096880

[4] McGuinness JE Trivedi MS Silverman T Marte A Mata J Kukafka R. Crew. Uptake of genetic testing for germline BRCA1/2 pathogenic variants in a predominantly Hispanic population. Cancer Genet. (2019) 235–6:72–6. 10.1016/j.cancergen.2019.04.063 31078448 PMC6625883

[5] Modell SM Allen CG Ponte A Marcus G. Cancer genetic testing in marginalized groups during an era of evolving healthcare reform. J Cancer Policy. (2021) 28:100275. 10.1016/j.jcpo.2021.100275 35559905 PMC8224823

[6] Modell SM Schlager L Allen CG Marcus G. Medicaid expansions: probing Medicaid's filling of the cancer genetic testing and screening space. Healthcare (Basel). (2022) 10:1066. 10.3390/healthcare10061066 35742117 PMC9223044

[7] Petelin L Trainer AH Mitchell G Liew D James PA. Cost-effectiveness and comparative effectiveness of cancer risk management strategies in BRCA1/2 mutation carriers: a systematic review. Genet Med. (2018) 20:1145–56. 10.1038/gim.2017.255 29323669

[8] Tuffaha HW Mitchell A Ward RL Connelly L Butler JRG Norris S. Cost-effectiveness analysis of germ-line BRCA testing in women with breast cancer and cascade testing in family members of mutation carriers. Genet Med. (2018) 20:985–94. 10.1038/gim.2017.231 29300376

[9] Lertwilaiwittaya P Roothumnong E Nakthong P Dungort P Meesamarnpong C Tansa-Nga W. Thai patients who fulfilled NCCN criteria for breast/ovarian cancer genetic assessment demonstrated high prevalence of germline mutations in cancer susceptibility genes: implication to Asian population testing. Breast Cancer Res Treat. (2021) 188:237–48. 10.1007/s10549-021-06152-4 33649982 PMC8233261

[10] Molina-Montes E Pérez-Nevot B Pollán M Sánchez-Cantalejo E Espín J Sánchez MJ. Cumulative risk of second primary contralateral breast cancer in BRCA1/BRCA2 mutation carriers with a first breast cancer: a systematic review and meta-analysis. Breast. (2014) 23:721–42. 10.1016/j.breast.2014.10.005 25467311

[11] Rebbeck TR Friebel T Lynch HT Neuhausen SL van 't Veer L Garber JE. Bilateral prophylactic mastectomy reduces breast cancer risk in BRCA1 and BRCA2 mutation carriers: the PROSE Study Group. J Clin Oncol. (2004) 22:1055–62. 10.1200/JCO.2004.04.188 14981104

[12] Rebbeck TR Kauff ND Domchek SM. Meta-analysis of risk reduction estimates associated with risk-reducing salpingo-oophorectomy in BRCA1 or BRCA2 mutation carriers. J Natl Cancer Inst. (2009) 101:80–7. 10.1093/jnci/djn442 19141781 PMC2639318

[13] Kauff ND Domchek SM Friebel TM Robson ME Lee J Judy E. Risk-reducing salpingo-oophorectomy for the prevention of BRCA1- and BRCA2-associated breast and gynecologic cancer: a multicenter, prospective study. J Clin Oncol. (2008) 26:1331–7. 10.1200/JCO.2007.13.9626 18268356 PMC3306809

[14] Domchek SM Friebel TM Singer CF Evans DG Lynch HT Isaacs C. Association of risk-reducing surgery in BRCA1 or BRCA2 mutation carriers with cancer risk and mortality. JAMA. (2010) 304:967–75. 10.1001/jama.2010.1237 20810374 PMC2948529

[15] Ingham SL Warwick J Buchan I Sahin S O'Hara C Moran A. Ovarian cancer among 8,005 women from a breast cancer family history clinic: no increased risk of invasive ovarian cancer in families testing negative for BRCA1 and BRCA2. J Med Genet. (2013) 50:368–72. 10.1136/jmedgenet-2013-101607 23539753

[16] Cameron D Piccart-Gebhart MJ Gelber RD Procter M Goldhirsch A de Azambuja E. 11 years' follow-up of trastuzumab after adjuvant chemotherapy in HER2-positive early breast cancer: final analysis of the HERceptin Adjuvant (HERA) trial. Lancet. (2017) 389:1195–205. 10.1016/S0140-6736(16)32616-2 28215665 PMC5465633

[17] Pan H Gray R Braybrooke J Davies C Taylor C McGale P. 20-year risks of breast-cancer recurrence after stopping endocrine therapy at 5 years. N Engl J Med. (2017) 377:1836–46. 10.1056/NEJMoa1701830 29117498 PMC5734609

[18] Bergfeldt K Rydh B Granath F Grönberg H Thalib L Adami HO. Risk of ovarian cancer in breast-cancer patients with a family history of breast or ovarian cancer: a population-based cohort study. Lancet. (2002) 360:891–4. 10.1016/S0140-6736(02)11023-3 12354469

[19] McGee J Giannakeas V Karlan B Lubinski J Gronwald J Rosen B. Risk of breast cancer after a diagnosis of ovarian cancer in BRCA mutation carriers: is preventive mastectomy warranted? Gynecol Oncol. (2017) 145:346–51. 10.1016/j.ygyno.2017.02.032 28314588

[20] Maajani K Jalali A Alipour S Khodadost M Tohidinik HR Yazdani K. The global and regional survival rate of women with breast cancer: a systematic review and meta-analysis. Clin Breast Cancer. (2019) 19:165–77. 10.1016/j.clbc.2019.01.006 30952546

[21] Chen C Xu Y Huang X Mao F Shen S Xu Y. Clinical characteristics and survival outcomes of patients with both primary breast cancer and primary ovarian cancer. Medicine (Baltimore). (2020) 99:e21560. 10.1097/MD.0000000000021560 32769897 PMC7593036

[22] World health organization. Thailand Source: Globocan 2020 International Agency for Research on Cancer. (2021). Available online at: https://gco.iarc.fr/today/data/factsheets/populations/764-thailand-fact-sheets.pdf (accessed April 18, 2022).

[23] Holland ML Huston A Noyes K. Cost-effectiveness of testing for breast cancer susceptibility genes. Value Health. (2009) 12:207–16. 10.1111/j.1524-4733.2008.00418.x 18647256

[24] Kwon JS Gutierrez-Barrera AM Young D Sun CC Daniels MS Lu KH. Expanding the criteria for BRCA mutation testing in breast cancer survivors. J Clin Oncol. (2010) 28:4214–20. 10.1200/JCO.2010.28.0719 20733129

[25] Petelin L Hossack L Shanahan M Mitchell G Liew D James PA. Cost-effectiveness of long-term clinical management of BRCA pathogenic variant carriers. Genet Med. (2020) 22:831–9. 10.1038/s41436-020-0751-3 31996782

[26] Guzauskas GF Garbett S Zhou Z Spencer SJ Smith HS Hao J. Cost-effectiveness of population-wide genomic screening for hereditary breast and ovarian cancer in the United States. JAMA Netw Open. (2020) 3:e2022874. 10.1001/jamanetworkopen.2020.22874 33119106 PMC7596578

[27] Green RC Berg JS Grody WW Kalia SS Korf BR Martin CL. ACMG recommendations for reporting of incidental findings in clinical exome and genome sequencing. Genet Med. (2013) 15:565–74. 10.1038/gim.2013.73 23788249 PMC3727274

